# Physicochemical Properties Determination of Recombinant Human Lysozyme and Its Effects on Intestinal Development in Mice

**DOI:** 10.3390/nu17233730

**Published:** 2025-11-28

**Authors:** Ruwei Liu, Qin An, Yunxia Zou, Zhuoxing Zhang, Qinyong Meng, Wentian Yue, Wenwen Dong, Yali Zhang

**Affiliations:** 1 Department of Food Science and Nutrition, College of Food Science and Nutritional Engineering, China Agricultural University, Beijing 100083, China; viviileo@126.com (R.L.);; 2 College of Food Science and Nutritional Engineering, China Agricultural University, Beijing 100083, China; anqin@cau.edu.cn (Q.A.);; 3 College of Biological Sciences, China Agricultural University, Beijing 100083, China; qymeng@cau.edu.cn

**Keywords:** recombinant human lysozyme, hen egg white lysozyme, *HT-29* cells, physicochemical properties, intestinal development

## Abstract

**Background/Objectives**: Breast milk lysozyme is crucial for infant intestinal health. The low breastfeeding rate has driven the investigation of alternatives like hen egg white lysozyme (HEWL) for infant formula supplementation. However, HEWL differs significantly from human lysozyme. This study aimed to systematically compare the functional efficacy of recombinant human lysozyme (rhLYZ) and HEWL to assess their suitability as formula supplements. **Methods**: The physicochemical properties (enzymatic activity, optimal pH, thermal stability) of rhLYZ and HEWL were analyzed. Biological functions were evaluated using *HT-29* intestinal cells for proliferation, differentiation, and protection against lipopolysaccharide-induced damage. In vivo effects on growth, intestinal morphology, and gene expression were assessed in a mouse pup model via transcriptomic analysis. Gut microbiota composition was also examined. **Results**: rhLYZ exhibited twice the enzymatic activity of HEWL, with an optimal pH of 6.0. In cellular models, rhLYZ enhanced intestinal epithelial differentiation at low concentrations. In vivo, rhLYZ supplementation significantly improved pup body weight, intestinal maturity, and villus-to-crypt ratios, outperforming HEWL. Transcriptomics revealed rhLYZ upregulated broad-spectrum antimicrobial peptides (e.g., *Defa*, *lactoferrin*) and immune-related genes, whereas HEWL induced a narrower antibacterial response and downregulated key defensins. Furthermore, rhLYZ significantly increased gut microbiota diversity and enriched beneficial butyrate-producing bacteria. **Conclusions**: rhLYZ more effectively mimics human milk lysozyme by promoting intestinal development, broad-spectrum immunity, and a balanced microbiota. HEWL shows a narrower functional profile. These findings provide a scientific basis for optimizing lysozyme selection in infant formula, highlighting the superior potential of rhLYZ.

## 1. Introduction

Lysozyme (LYZ), an alkaline hydrolase widely distributed in biological fluids such as saliva, tears, and milk, serves as a crucial component of the innate immune defense system [1]. In vertebrates, the C-type lysozyme is predominant. Human lysozyme (hLYZ) and hen egg white lysozyme (HEWL) are classic C-type model proteins, valued for their physiological relevance and accessibility, respectively [2,3]. Despite both belonging to the C-type, they share only 60% primary structure homology, yet their tertiary structures—featuring four α-helices, three β-sheets, and four disulfide bonds—are highly conserved, with active sites (*Glu35* and *Asp53*) that catalyze the hydrolysis of bacterial peptidoglycan (PG) [4,5,6].

The antibacterial function of lysozyme involves a dual mechanism: enzymatic hydrolysis of PG, effective against Gram-positive bacteria, and a cationic antimicrobial peptide-like action that can penetrate Gram-negative bacterial membranes [7,8,9]. Pathogens counteract this via PG modifications or specific inhibitors [9]. Beyond direct bactericidal activity, hLYZ plays a significant immunomodulatory role in the gut, exhibiting both pro-inflammatory potential through released pathogen-associated molecular patterns (PAMPs) and, more importantly, potent anti-inflammatory and protective effects. These include LPS binding, enhancement of epithelial barrier integrity, and modulation of immune cell functions and cytokine secretion, thereby strengthening overall mucosal immunity [10,11,12].

In infant nutrition, the concentration of human milk lysozyme (e.g., 5.01 g/L in mature milk) vastly exceeds that in bovine or goat milk, underpinning its critical role in newborn defense against pathogens, promotion of beneficial *Bifidobacterium* colonization, and support of intestinal barrier and immune development [1,13,14,15,16]. Consequently, lysozyme supplementation is a key strategy for the “humanization” of infant formula. Currently, in experimental research and development of such ‘humanized’ formulas, hen egg white lysozyme (HEWL) is the primary candidate under consideration due to cost and scalability constraints, despite hLYZ offering superior biocompatibility and potentially greater efficacy. The limited natural sources of hLYZ have been overcome by recombinant technology (rhLYZ), which produces a protein highly consistent with native hLYZ in structure and function [17,18,19,20,21].

While HEWL has been the predominant candidate in experimental studies of infant formula supplementation, rhLYZ emerges as a novel, more homologous alternative. This raises critical questions: despite both being C-type lysozymes, what are the specific differences in their physicochemical properties? More importantly, do they exert equivalent effects on neonatal intestinal development, immune programming, and microbial colonization? Although rhLYZ shows promise in medical [22,23] and food applications, including as a dairy preservative and microbiome modulator [24], a direct and systematic comparison of its functional benefits for infant gut health against the commonly studied HEWL is lacking [25,26,27,28].

Therefore, our work was designed to directly address these questions. We systematically compared the physicochemical and functional properties of rhLYZ and HEWL, with a focus on their impacts on intestinal development and microbiota. Our findings demonstrate that rhLYZ significantly outperforms HEWL in promoting intestinal morphogenesis, establishing broad-spectrum immune defenses and fostering a balanced microbial ecosystem, thus offering a superior functional profile for application in infant nutrition.

## 2. Materials and Methods

### 2.1. Materials

rhLYZ with a purity of 50% (SDS-PAGE analysis) was expressed through a bovine mammary gland bioreactor system by the State Key Laboratory of Biotechnology, China Agricultural University. HEWL with 95% purity (SDS-PAGE analysis) was purchased from Sigma Aldrich (St. Louis, MO, USA) (Product No. L6876). LPS was purchased from Sigma Aldrich (Product No. L2880).

### 2.2. Recombinant Human Lysozyme (rhLYZ) Purification

The rhLYZ crude extract was fully dissolved in 20 mM phosphate buffer (pH 7.2) at a concentration of 15 mg/mL, filtered through a 0.22 μm membrane, and purified using an ÄKTA pure 10 system (Cytiva, Marlborough, MA, USA). A 10 mL sample (15 mg/mL) was loaded onto a 16/60 Superdex 75 prep-grade column (Taidu biotech, Suzhou, China) at an elution flow rate of 0.5 mL/min, with rhLYZ-containing fractions collected. These fractions were concentrated and desalted using 3 kDa cutoff ultrafiltration tubes (Millipore (Burlington, MA, USA) Amicon Ultra-15) via high-speed refrigerated centrifugation (12,000× *g*, 15 °C, 15 min). The resulting concentrate was lyophilized, and the obtained powder was verified for rhLYZ purity by SDS-PAGE analysis [29].

### 2.3. Lysozyme Activity and Physicochemical Characterization

Lysozyme activity was determined turbidimetrically using *Micrococcus lysodeikticus* (CGMCC, Beijing, China) as a substrate, following the method of Shugar [30]. One unit of enzyme activity (U) was defined as a decrease in A450 of 0.001 per minute. Specific activity was expressed as U per mg of protein (U/mg).

The optimal temperature for enzymatic activity was assessed by measuring activity across a range of temperatures (25, 37, 50, 65, 80, and 100 °C) in 66 mM potassium phosphate buffer (pH 6.24). Thermal stability was evaluated by incubating enzyme solutions (100 μg/mL) at 50, 65, 80, and 100 °C, with residual activity measured at predetermined time intervals over 60 min. The optimal pH was determined by pre-incubating enzymes (10 μg/mL) in buffers at varying pH (2.0–11.0) for 10 min, followed by immediate activity measurement. pH stability was assessed by incubating enzymes in the same range of buffers for 20 min at 25 °C, after which residual activity was measured at pH 7.0. All assays were performed in triplicate.

### 2.4. Fluorescent Protein Localization

2 g Sephadex G-100 gel (Solarbio (Beijing, China), S8170) was swollen in distilled water for ≥24 h. The swollen gel was packed into a vertical glass column (15 × 100 mm) pre-filled with 3/4 volume PBS by gentle tapping to eliminate air pockets, maintaining a 3 cm headspace below the column top with continuous PBS coverage. For protein labeling, 25 mg rhLYZ or HEWL was dissolved in 5 mL 0.1 M sodium carbonate buffer. Under constant stirring, 250 μL FITC solution was added dropwise, followed by 8 h dark incubation at 4 °C. The reaction was terminated with 50 mM NH4Cl (2 h, 4 °C), then supplemented with 0.1% xylene cyanol and 5% glycerol. Post-equilibration (PBS drained to gel level), FITC-labeled proteins were loaded in darkness. Elution with PBS was performed under light-protected conditions while maintaining liquid sealing. Initial yellow fractions (fluorescent proteins) were collected and quantified using a BCA assay kit [31]. For cellular imaging, HT-29 cells (Procell system, Wuhan, China) were seeded in 24-well plates at 2.5 × 10^5^ cells/well. After 24 h culture, cells were treated with 500 μg/mL fluorescently labeled rhLYZ/HEWL for 48 h and imaged by fluorescence inverted microscopy.

### 2.5. Functional Cell-Based Assays: Proliferation, Differentiation, and Cytoprotection

A series of in vitro assays were conducted using *HT-29* cells to comprehensively evaluate the biological activities of rhLYZ and HEWL. HT-29 cells were cultured in McCoy’s 5A medium supplemented with 10% fetal bovine serum (FBS) and 1% penicillin–streptomycin. For the proliferation assay, cells were seeded in 96-well plates at 5 × 10^3^ cells/well. After 24 h of adherence, they were treated with a concentration gradient of rhLYZ or HEWL (0, 1, 10, 100, 500, or 1000 μg/mL) for 24, 48, and 72 h. This assay included an untreated control (0 μg/mL), a negative control (500 μg/mL BSA), and a blank control (cell-free wells). Cell proliferation was quantified using a CCK-8 kit from LABLEAD Inc. (Beijing, China). To assess the induction of differentiation, cells were seeded at a high density (1 × 10^5^ cells/well) and cultured to confluence before being treated with the same compound series (0–1000 μg/mL) for 14 or 21 days. The culture medium was refreshed every other day with new medium containing the same concentration of lysozyme throughout the 14 or 21-day treatment period. Alkaline phosphatase activity, a differentiation marker, was then measured. Furthermore, the cytoprotective effect against LPS-induced damage was investigated. Cells were pretreated with rhLYZ, HEWL, or BSA (500 μg/mL) for 24 h prior to a 24 h challenge with LPS (150 μg/mL). Experimental groups consisted of Blank, Untreated, LPS-injured Model, and Treatment/Control groups. Ultimately, cell viability in this assay was also determined using the CCK-8 method.

### 2.6. Animal

Ten male and twenty female C57BL/6J mice (8 weeks old, body weight 24 ± 2 g) were procured from Beijing Vital River Laboratory Animal Technology Co., Ltd. (Beijing, China) and maintained under specific pathogen-free (SPF) conditions. These animals were used for breeding, yielding 24 neonatal C57BL/6J mice that were also raised under SPF conditions. The experimental unit was defined as a single neonatal mouse. Neonates from multiple litters were randomly allocated into three groups (CON, rhLYZ, and HEWL) receiving daily oral gavage for 18 days: 0.85% NaCl (CON group), 100 mg/kg recombinant human lysozyme (rhLYZ group), or 100 mg/kg hen egg white lysozyme (HEWL group), This dose aligns with established preclinical models (e.g., 90–100 mg/kg in pigs [32]; 300 mg/kg in murine studies [33], with ad libitum nursing maintained throughout. The sample size was determined based on established protocols from previous literature in similar experimental settings. Daily food intake was monitored and remained comparable across groups. All animals were housed under SPF conditions in accordance with national standards for laboratory animal requirements (GB14925-2010) governing environmental and housing conditions. All procedures were approved by the Institutional Animal Care and Use Committee of China Agricultural University (issue No. AW70304202-4-1). Terminal procedures were performed under sodium pentobarbital anesthesia (100 mg/kg, intraperitoneal). Humane endpoints were established for this study. Animals were monitored twice daily for signs of severe distress, including labored breathing indicative of aspiration, profound lethargy, or rapid weight loss (>20%). Any animal meeting these criteria would have been euthanized immediately. The mortalities that occurred during gavage procedures represented the primary endpoint encountered.

### 2.7. Animal Tissue Sample Collection

The small intestine and colon were weighed, with small intestine length recorded. Cecal contents were collected separately and flash frozen. Segments of the small intestine (duodenum, jejunum, ileum) and colon were fixed in 4% paraformaldehyde solution for subsequent sectioning. The remaining tissues were immediately flash-frozen in liquid nitrogen and stored at −80 °C.

### 2.8. Maltase-Lactase Activity Assay

Small intestinal tissues were homogenized in ice-cold saline at a 1:9 (*w*/*v*) tissue-to-solution ratio using an ice-water bath. The homogenate was centrifuged at 5000 rpm (4 °C) for 10 min to obtain a 10% mucosal supernatant. Maltase and lactase activities were measured in the 10% mucosal homogenate using commercial assay kits (Jiancheng Bioengineering, Nanjing, China). Protein concentration was determined with a BCA protein assay kit (Beyotime Biotechnology, Shanghai, China), followed by calculation of specific enzyme activities.

### 2.9. Histological and Morphometric Analysis

Paraffin-embedded tissue blocks were sectioned at 4 μm thickness using a Leica microtome (Wetzlar, Germany). For each experimental group (*n* = 5–6 mice), three sections per mouse were prepared, with at least four fields analyzed per section. Sections underwent automated deparaffinization followed by dual staining protocols: Hematoxylin & Eosin (H&E) using a commercial kit (Solarbio, Beijing, China) and Lendrum’s phloxine-tartrazine stain (Delta, Xi’an, China), Lendrum’s phloxine-tartrazine stain was used specifically for the identification and quantification of Paneth cells. Quantitative morphometric analysis was performed using ImageJ software (version 1.53t; National Institutes of Health, Bethesda, MD, USA).

### 2.10. Quantitative Real-Time Polymerase Chain Reaction (qPCR)

Intestinal tissue samples (duodenum, jejunum, and ileum) from all experimental groups were processed for gene expression analysis. To ensure objective assessment, all samples were systematically coded and analyzed in a single-blind manner by an external research facility. Frozen tissues stored at −80 °C were homogenized in TRIzol reagent (TIANGEN, Beijing, China) for total RNA extraction. RNA concentration and purity were verified using a NanoDrop 1000A spectrophotometer (Thermo Fisher Scientific, Walltham, MA, USA). Reverse transcription was performed with 1 μg total RNA in a 20 μL reaction volume using All-In-One 5X RT MasterMix (Lablead, Beijing, China). Quantitative real-time PCR was conducted on a StepOne Real-Time PCR System (Lablead, China) with Power SYBR Green PCR Master Mix (Applied Biosystems, Waltham, MA, USA), strictly following the manufacturers’ protocols.

### 2.11. Transcriptome Sequencing

Total RNA was extracted using TRIzol reagent with DNase I digestion to remove genomic DNA. RNA quality was assessed through agarose gel electrophoresis, Nanodrop spectrophotometry, and Agilent (Santa Clara, CA, USA) 2100 Bioanalyzer (RIN ≥ 7). All subsequent procedures were performed under single-blind conditions with samples coded to conceal group identities. Strand-specific libraries were prepared using the Illumina (San Diego, CA, USA) TruSeq Stranded mRNA Kit and sequenced on the NovaSeq 6000 platform (PE150, ≥10 Gb per sample; *n* = 3 per group). Raw data were processed using FastQC and Trimmomatic, followed by alignment to the reference genome with STAR/HISAT2. Gene expression was quantified using FeatureCounts, and differential expression analysis was performed with DESeq2/edgeR (|log2FC| ≥ 1, FDR < 0.05). Sample group identities were revealed only after completion of all computational analyses. Functional enrichment analysis was conducted using GO and KEGG databases.

### 2.12. Intestinal Microbial Diversity Analysis

NanoDrop spectrophotometry. All experimental procedures were conducted under single-blind conditions with coded samples. High-fidelity DNA polymerase was employed to amplify target regions using the extracted DNA from the flash-frozen cecal content samples as template, followed by purification of PCR products with AMPure XP Beads. Purified amplicons were ligated to Illumina sequencing adapters through Index PCR for library construction. The resulting libraries underwent additional purification with AMPure XP Beads, with fragment size distribution and concentration quantified using either the Agilent 2100 Bioanalyzer or Qubit fluorometer. High-throughput paired-end sequencing (PE250 or PE300) was performed on Illumina NovaSeq 6000 or MiSeq platforms, generating 50–100 K reads per sample to ensure comprehensive microbial community coverage (*n* = 4 per group). Sample group identities remained concealed throughout the sequencing and initial data processing phases. Quantitative data were analyzed using IBM SPSS Statistics 26 and visualized with GraphPad Prism 8. Results are expressed as mean ± SD. For two-group comparisons, Student’s *t*-test was applied with significance thresholds denoted as * *p* < 0.05, ** *p* < 0.01, and *** *p* < 0.001. Multi-group analyses utilized one-way, two-way, or three-way ANOVA followed by post hoc Duncan’s tests. The blinding codes were only broken after completion of all laboratory analyses and prior to statistical evaluation.

### 2.13. Statistical Analysis

Quantitative data were analyzed using IBM SPSS Statistics 26 and visualized with GraphPad Prism 8. All results are presented as mean ± SD. The criteria for excluding animals and data points were pre-established. These included death due to gavage procedures and statistical outliers identified in intestinal development analyses using Grubbs’ test (α = 0.05). In the control group, two suckling mice were excluded from all analyses following gavage-related mortality. The HEWL group initially contained eight animals; one was excluded due to fatal gavage injury and an additional animal was removed as a statistical outlier specifically in the intestinal development analysis. Similarly, the rhLYZ group began with eight animals, losing one to gavage complications and another identified as a statistical outlier in intestinal development assessment. Consequently, the final sample sizes for analysis were *n* = 6 for the three groups. Statistical comparisons between two groups were performed using Student’s *t*-test, while multi-group comparisons employed one-way, two-way, or three-way ANOVA followed by Duncan’s post hoc tests. Significance levels were designated as * *p* < 0.05, ** *p* < 0.01, and *** *p* < 0.001.

## 3. Results

### 3.1. Recombinant Human Lysozyme (rhLYZ) Purification

**Figure 1:**
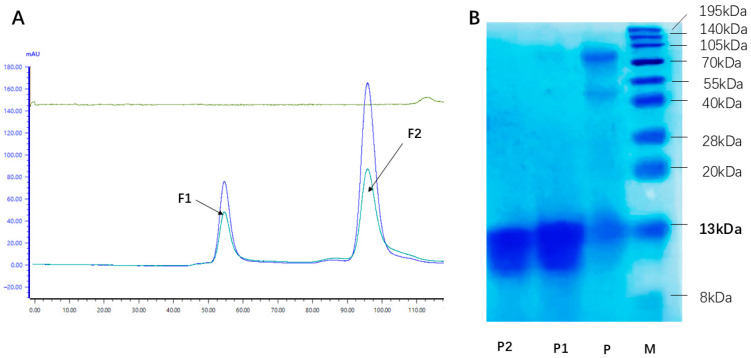
Gel filtration chromatography and SDS-PAGE analysis of purified rhLYZ. (**A**) Purification of rhLYZ by gel filtration chromatography. FI represents the elution peak of contaminating proteins, and F2 corresponds to the elution peak of the rhLYZ fraction. (**B**) Identification by 15% SDS-PAGE staining (the elution peak F2 from A was concentrated and loaded; M: Marker; P: Crude rhLYZ; P1: Purified rhLYZ; P2: HEWL).

The rhLYZ crude product was purified via gel filtration chromatography, during which peak P2 was identified as the rhLYZ elution peak, as confirmed by SDS-PAGE analysis (Figure 1A). Following concentration and desalting, the final product was obtained. The purity of this final product was quantified at 96.2% by densitometric analysis of a Coomassie Brilliant Blue-stained SDS-PAGE gel using Quantity One software (version 4.6.9; Bio-Rad Laboratories, Inc., Hercules, CA, USA), where purity was calculated as the integrated intensity ratio of the target rhLYZ band to all bands in the lane (Figure 1B). All rhLYZ used in subsequent experiments was derived from this purification process.

### 3.2. Evaluation of Physicochemical Properties of rhLYZ and HEWL

**Figure 2:**
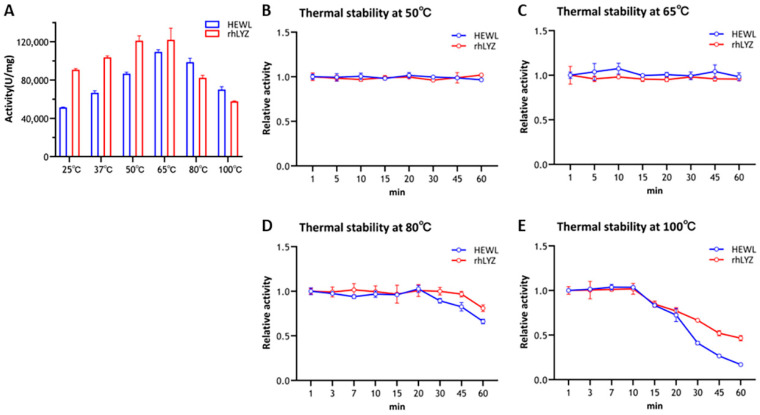
Optimal Temperature of rhLYZ and HEWL and thermal stability of rhLYZ and HEWL at 50 °C, 65 °C, 80 °C, and 100 °C. The results are expressed as SD ± mean, *n* = 3. (**A**) activity; (**B**) thermal stability at 50 °C; (**C**) thermal stability at 65 °C; (**D**) thermal stability at 80 °C; (**E**) thermal stability at 100 °C.

Optimal temperature and themostability. Experimental results demonstrated that purified rhLYZ exhibited twice the lytic activity against Micrococcus luteus compared to HEWL at 25 °C (Figure 2A). Both enzymes displayed a consistent bell-shaped activity profile from 25 °C to 100 °C, peaking at 65 °C. rhLYZ showed higher activity than HEWL between 25–65 °C, whereas HEWL outperformed rhLYZ from 65–100 °C: HEWL retained 140% of its 25 °C activity at 100 °C, while rhLYZ activity declined to 64%. Nevertheless, rhLYZ maintained superior overall enzymatic activity. Thermal stability assays revealed no significant activity loss after 60 min incubation at 50–65 °C for either enzyme. At 80 °C, HEWL activity decreased to 66% after 60 min, contrasting with rhLYZ retention of 81% activity. Following 100 °C treatment for 60 min, HEWL retained only 17% activity versus rhLYZ 47%, conclusively demonstrating rhLYZ significantly enhanced thermostability relative to HEWL (Figure 2B–E).

**Figure 3:**
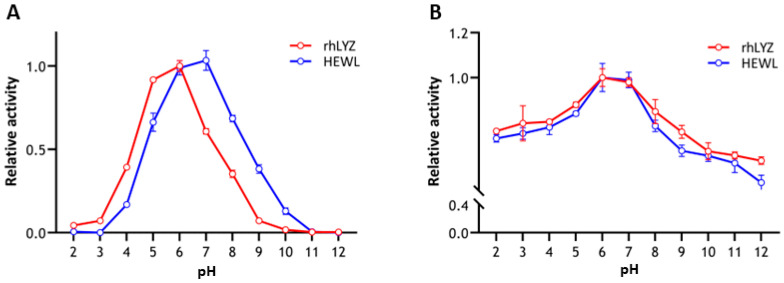
(**A**) Optimal pH of rhLYZ and HEWL; (**B**) pH stability of rhLYZ and HEWL. The results are expressed as SD ± mean, *n* = 3.

Optimal pH and pH stability. Experimental results revealed distinct optimal pH values for rhLYZ and HEWL, with rhLYZ exhibiting peak enzymatic activity at pH 6.0 while HEWL showed maximum activity at pH 7.0. Both enzymes underwent dramatic activity reduction under extreme pH conditions (Figure 3A). Further investigation demonstrated robust pH stability for both lysozymes within the pH 2.0–12.0 range, where at pH 2.0 HEWL retained 72% relative activity versus rhLYZ’s 76%, and at pH 12.0 HEWL maintained 53% activity compared to rhLYZ’s 63%. These findings collectively indicate that both enzymes possess considerable acid-base tolerance, with rhLYZ consistently exhibiting marginally superior stability across the tested pH spectrum (Figure 3B).

### 3.3. Effects of rhLYZ and HEWL on HT29 Cells

**Figure 4:**
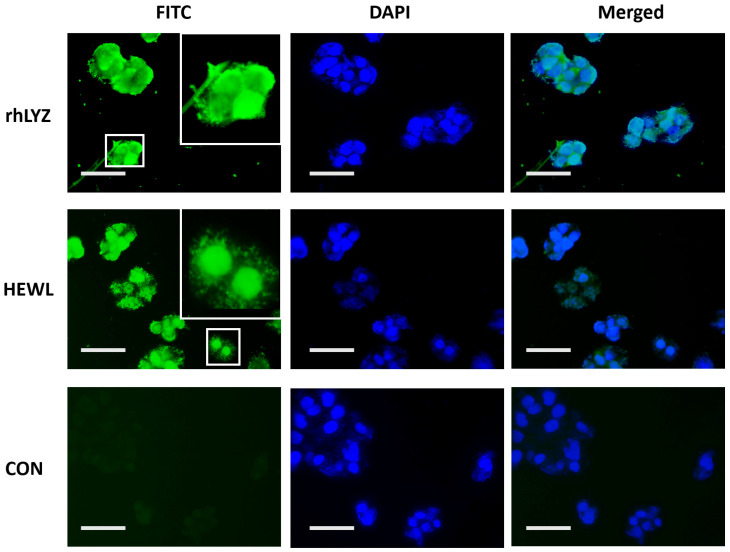
Fluorescently Labeled Lysozyme Acting on HT-29 Cells. FITC-labeled lysozyme was used to treat HT-29 cells, with FITC employed to label rhLYZ and HEWL, and DAPI for nuclear staining (scale bar = 20 μm).

Fluorescent protein localization. Experimental results in Figure 4 show that after two days of incubation, partial FITC fluorescence signals from both rhLYZ and HEWL completely overlapped with DAPI signals. The control group without lysozyme exhibited minimal fluorescence, demonstrating that both lysozymes can enter cells and distribute in both cytoplasmic and nuclear compartments.

**Figure 5:**
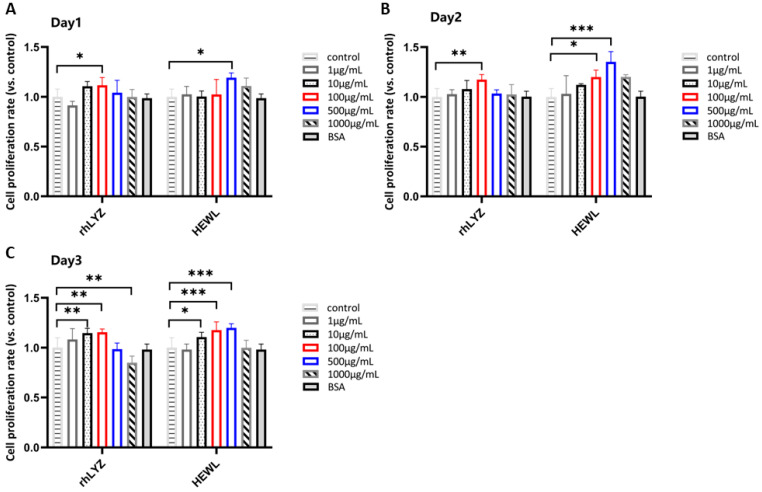
Effects of rhLYZ and HEWL on HT-29 cell proliferation. (**A**) Changes in cell proliferation rate after 1-day incubation with rhLYZ or HEWL; (**B**) Changes in cell proliferation rate after 2-day incubation with rhLYZ or HEWL; (**C**) Changes in cell proliferation rate after 3-day incubation with rhLYZ or HEWL. The results are expressed as mean ± SD, *n* = 5. The significance of the differences was estimated by one-way ANOVA, * *p* < 0.05, ** *p* < 0.01, *** *p* < 0.001.

Cell Proliferation. Results showed that treating HT-29 cells with a concentration gradient (1–1000 μg/mL) for 24 h, 48 h, and 72 h revealed proliferation-promoting effects for both rhLYZ and HEWL, though with differing effective concentrations and time dependencies. Based on the results over 72 h, the optimal proliferation-promoting concentration was 100 μg/mL for rhLYZ and 500 μg/mL for HEWL. At the high concentration (1000 μg/mL), the proliferative effects of both diminished, with rhLYZ being more prone to exhibit inhibitory effects (Figure 5A–C).

**Figure 6:**
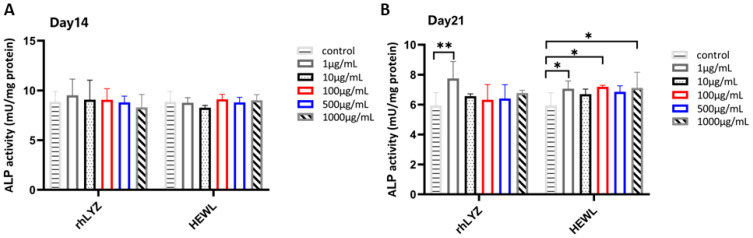
Effects of rhLYZ and HEWL on alkaline phosphatase (ALP) activity in HT-29 cells. (**A**) Changes in ALP activity after 14-day incubation with rhLYZ or HEWL; (**B**) Changes in ALP activity after 21-day incubation with rhLYZ or HEWL. The results are expressed as mean ± SD, *n* = 5. The significance of the differences was estimated by one-way ANOVA, * *p* < 0.05, ** *p* < 0.01.

Cell Differentiation. Cells were treated with rhLYZ or HEWL (1, 10, 100, 500, or 1000 μg/mL), with controls cultured in lysozyme-free McCoy’s 5A medium. Results revealed no significant differences in alkaline phosphatase (ALP) activity between treatment groups and controls at day 14. Prolonged intervention (21 days) induced more significant pro-differentiation effects: rhLYZ efficiently induced differentiation even at a low concentration (1 μg/mL), whereas HEWL required higher concentrations (≥100 μg/mL) and exhibited weaker efficacy. This suggests that rhLYZ demonstrates greater efficiency and potential in regulating intestinal epithelial differentiation (Figure 6A,B).

**Figure 7:**
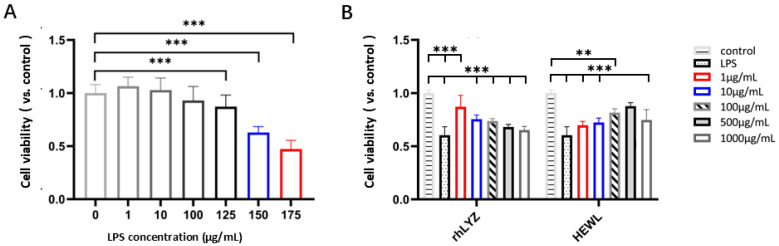
Protective effects of rhLYZ and HEWL on LPS-injured HT-29 cells. (**A**) Effect of LPS on HT-29 cell viability; (**B**) Protective effects of rhLYZ and HEWL on LPS-injured HT-29 cells. The results are expressed as mean ± SD, *n* = 5. The significance of the differences was estimated by one-way ANOVA, ** *p* < 0.01, *** *p* < 0.001.

Damage Protection. Treatment with LPS (1–175 μg/mL, 24 h) induced a dose-dependent decrease in cell viability. Specifically, 150 μg/mL LPS reduced the viability rate to 63% of the control level (Figure 7A). This concentration was selected to establish a model for evaluating the protective effects of lysozymes. Cells were pretreated with rhLYZ or HEWL (0–1000 μg/mL) for 24 h, followed by LPS challenge (150 μg/mL, 24 h). rhLYZ pretreatment (1–10 μg/mL) significantly enhanced cell viability. At 1 μg/mL, rhLYZ fully restored the viability rate to normal levels (comparable to the undamaged control). However, its protective effect diminished with increasing concentration. Conversely, HEWL significantly enhanced viability within 100–1000 μg/mL. At 500 μg/mL, HEWL restored viability to normal levels. Its protective effect generally increased with concentration, though a slight decline was observed at 1000 μg/mL. In summary, rhLYZ provides potent cytoprotection at low concentrations (1 μg/mL), while HEWL requires higher concentrations (≥100 μg/mL) and exhibits a distinct protective profile (Figure 7B).

### 3.4. rhLYZ Can More Significantly Promote Early Intestinal Development in 18-Day-Old Mice

**Figure 8:**
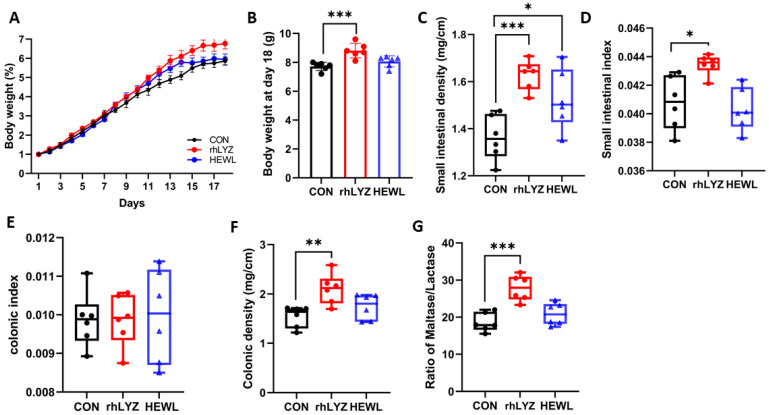
Intestinal development status in18-day-old mice of the rhLYZ, HEWL and CON groups. (**A**) Body weight changes (days 1–18); (**B**) Day 18 body weight; (**C**) Small intestinal density; (**D**) Small intestinal index; (**E**) Colonic density; (**F**) Colonic index; (**G**) Maltase/lactase activity in duodenum, jejunum and ileum (*n* = 6). The results are expressed as mean ± SD, *n* = 6. The significance of the differences was estimated by one-way ANOVA, * *p* < 0.05, ** *p* < 0.01, *** *p* < 0.001.

Body weight measurements of 18-day-old mice (Figure 8A,B) revealed significantly greater weight gain in the rhLYZ group compared to both control and HEWL groups by day 18. Morphological analyses demonstrated that rhLYZ administration substantially increased small intestinal density, organ coefficient index, and colonic density relative to controls (Figure 8C–F). While HEWL treatment elevated small intestinal density (Figure 8C), it showed no significant effects on other parameters. Regarding intestinal maturation biomarkers, the maltase/lactase activity ratio—a key indicator of small intestinal maturation where maltase activity rises and lactase declines during development—was significantly enhanced in duodenal, jejunal, and ileal segments of rhLYZ-treated mice (100 mg/kg) versus control and HEWL groups (Figure 8G).

**Figure 9:**
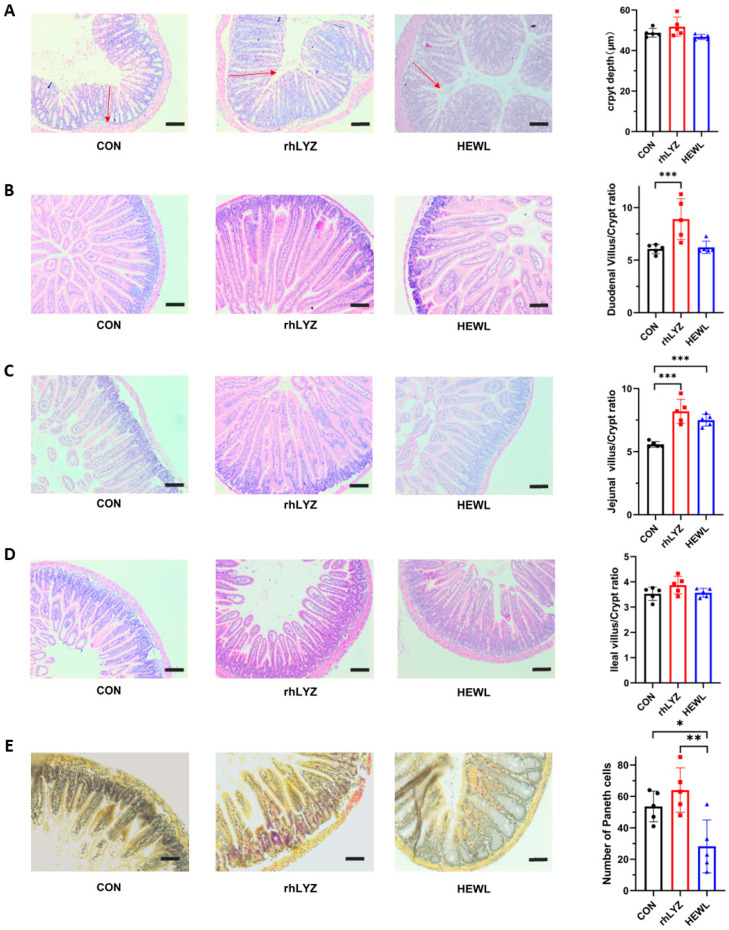
Intestinal sections in neonatal mice for the rhLYZ, HEWL, and CON groups. (**A**) Colon crypt depth; the red headed arrow indicates the crypt length. (**B**) Duodenal villus-to-crypt ratio; (**C**) Jejunal villus-to-crypt ratio; (**D**) Ileal villus-to-crypt ratio; (**E**) Paneth cell count in ileum (scale bar = 50 μm). The results are expressed as mean ± SD, *n* = 5. The significance of the differences was estimated by one-way ANOVA, * *p* < 0.05, ** *p* < 0.01, *** *p* < 0.001.

Hematoxylin-eosin (HE) staining analysis revealed no significant differences in colonic crypt depth among the three groups (Figure 9A). The rhLYZ-treated group exhibited a significantly higher duodenal villus-to-crypt ratio than both the control and HEWL groups (Figure 9B). In the jejunum, both rhLYZ and HEWL groups showed elevated villus-to-crypt ratios compared to controls, whereas no intergroup differences were observed in the ileum (Figure 9C,D). Quantitative assessment of Paneth cell numbers in the ileal segments demonstrated that the HEWL group had significantly fewer Paneth cells than both the rhLYZ group and control group (Figure 9E).

### 3.5. HEWL Induces an Immunosuppressive State in 18-Day-Old Mice by Inhibiting Paneth Cell Development in the Small Intestine

**Figure 10:**
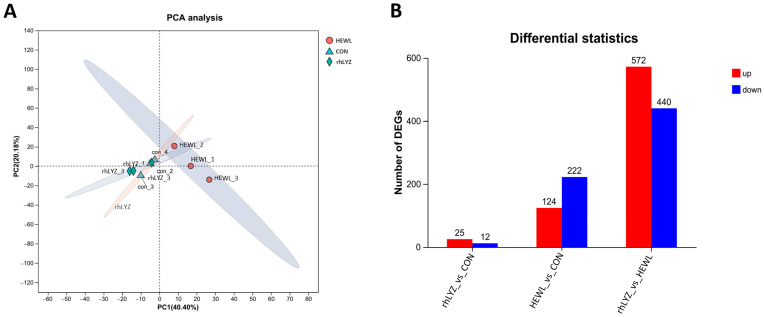
PCA Analysis of Intestinal Transcriptome and Differential Gene Expression Analysis in neonatal mouse of the rhLYZ, HEWL and CON groups; (**A**) PCA analysis of the intestinal. (**B**) Counts of differentially expressed genes (DEGs) in 18-day-old mice (*n* = 3).

As shown in Figure 10A, all three sample groups exhibited distinct separation, with the HEWL group demonstrating substantial differences compared to both the rhLYZ and control (CON) groups. Differential expression analysis using edgeR further revealed only 37 differentially expressed genes (DEGs) between the rhLYZ and control groups, whereas 346 DEGs were identified between the HEWL and control groups. Notably, 1012 DEGs were detected between the rhLYZ and HEWL groups (Figure 10B). Consequently, 18-day rhLYZ gavage administration induced minimal alterations in small intestinal gene expression in mice, while HEWL significantly remodeled the transcriptional landscape. The markedly higher number of DEGs between rhLYZ and HEWL underscores their divergent biological effects.

**Figure 11:**
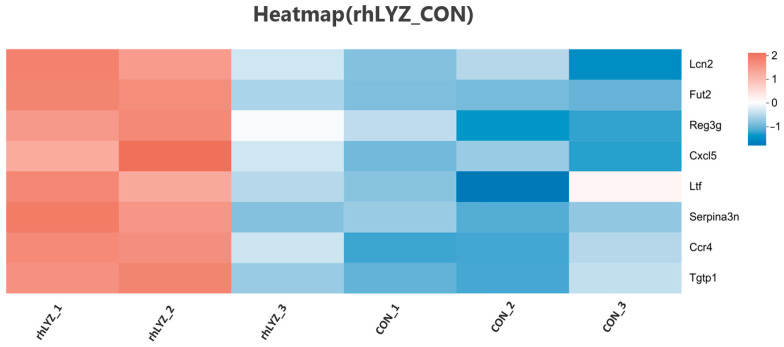
Analysis of immune-related differentially expressed gene sets in the neonatal mouse intestinal transcriptome of the rhLYZ vs. CON.

**Figure 12:**
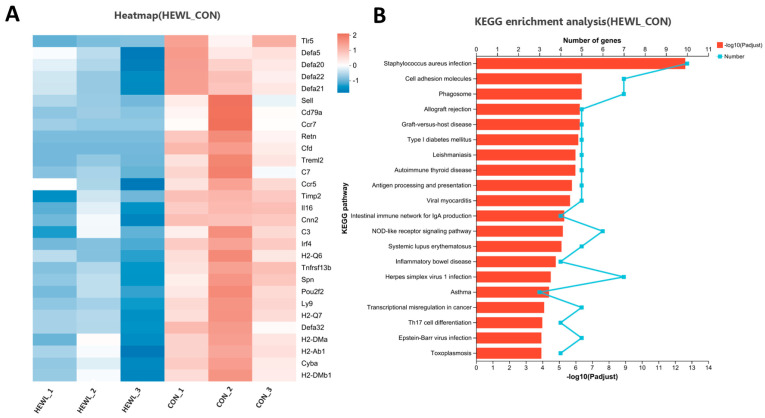
Analysis of immune-related differentially expressed gene sets in the neonatal mouse intestinal transcriptome of the HEWL vs. CON. (**A**) Cluster analysis of immune-related DEGs for HEWL vs. CON; The color scale represents normalized expression levels, with red indicating up-regulation and blue indicating down-regulation in the HEWL group compared to CON; (**B**) KEGG functional enrichment analysis of immune-related DEGs for HEWL vs. CON; The color gradient of the bars corresponds to the statistical significance of the enrichment (−log_10_(*p*-value)); *n* = 3.

**Figure 13:**
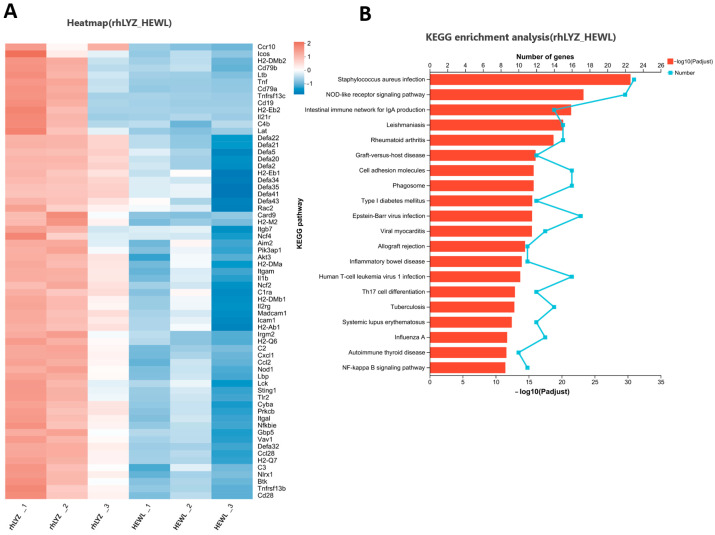
Analysis of immune-related differentially expressed gene sets in the neonatal mouse intestinal transcriptome of the rhLYZ vs. HEWL. (**A**) Cluster analysis of immune-related DEGs for rhLYZ vs. HEWL; (**B**) KEGG functional enrichment analysis of immune-related DEGs for rhLYZ vs. HEWL. *n* = 3.

Compared to the control group, the rhLYZ group showed only 37 DEGs, with all 8 immune-related genes (*Lcn2*, *Fut2*, *Reg3g*, *Cxcl5*, *Ltf*, *Serpina3n*, *Ccr4* and *Tgtp1*) significantly upregulated (Figure 11). In contrast, the HEWL group exhibited 346 DEGs versus controls, where 23 out of 28 immune-related genes—specifically innate immunity genes (e.g., *Tlr5*, Defensin family)—along with all 5 adaptive immunity genes were significantly downregulated (Figure 12A). Between rhLYZ and HEWL groups (Figure 13A), 1012 DEGs were identified, with all 65 immune-related genes upregulated—including 37 adaptive immunity genes (e.g., MHC class II molecules, *Ccl28*) and 37 innate immunity genes (e.g., α-defensins, complement components). These genes were highly enriched in pathways such as Staphylococcus aureus infection and NOD-like receptor signaling, indicating that rhLYZ synergistically activates immune responses by enhancing antigen presentation, recruiting mucosal immunity, and promoting antimicrobial peptide secretion (Figure 12B and Figure 13B). Conversely, HEWL treatment resulted in broad downregulation of both innate and adaptive immunity genes, inducing an immunosuppressive state.

**Figure 14:**
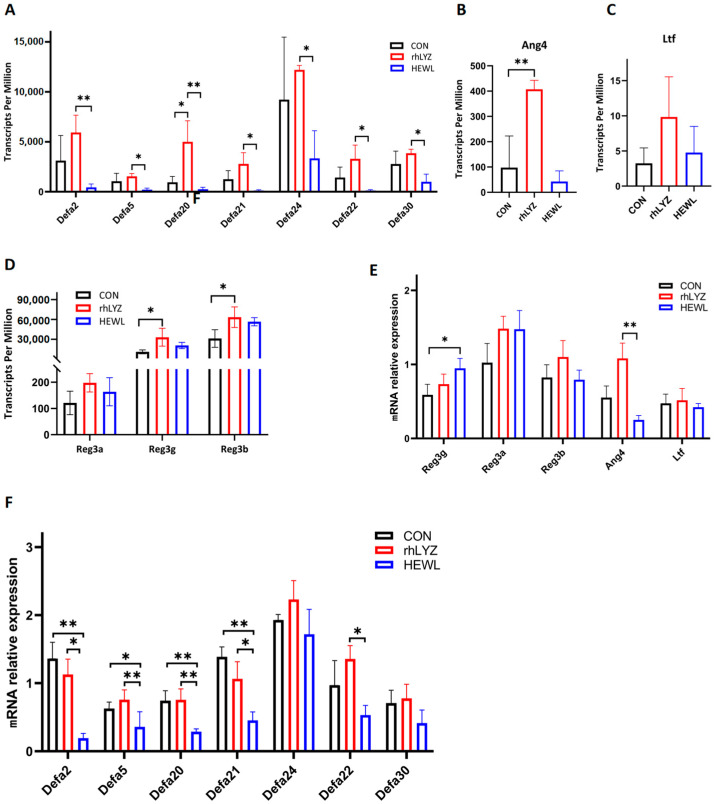
Expression Levels of Antimicrobial Peptides in the Intestinal Transcriptomes of the rhLYZ, HEWL and CON groups. (**A**) Defensin (*Defa*) expression; (**B**) *Ang4* expression; (**C**) *Ltf* expression; (**D**) *Reg3* family expression; (**E**,**F**) mRNA expression. Antimicrobial proteins in the intestine were measured by RNA Sequencing (**A**–**D**) and Q-PCR (**E**,**F**). The results are expressed as mean ± SD, *n* = 3. The significance of the differences was estimated by one-way ANOVA, * *p* < 0.05, ** *p* < 0.01.

Among key intestinal antimicrobial proteins,α-defensins (Defa family) and *Ang4* secreted by Paneth cells play a central antibacterial role by directly disrupting pathogen membrane structures (Figure 14A–C), while *Reg3* family proteins produced by goblet cells exhibit direct bactericidal activity (Figure 14D). Transcriptomic and qPCR data revealed that compared to the rhLYZ group, HEWL treatment significantly reduced the expression of Paneth cell-specific antimicrobial proteins (α-defensins and *Ang4*), whereas *Reg3* family protein expression showed a modest increase (Figure 14E,F). Combined with histological observations of markedly decreased Paneth cell numbers in the HEWL group, these findings indicate that HEWL delays the developmental progression of Paneth cells in the small intestine of 18-day-old mice, thereby impairing their critical antimicrobial defense functions.

### 3.6. Effects of rhLYZ and HEWL on Intestinal Microbial Composition

**Figure 15:**
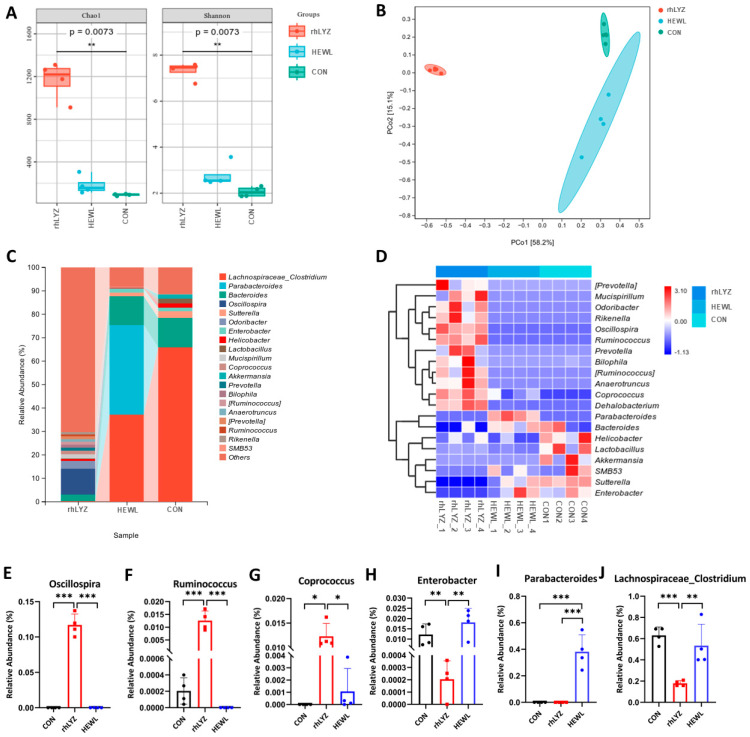
Effects of rhLYZ and HEWL on developing gut microbiota composition. (**A**) α-diversity (Chao1, Shannon); (**B**) β-diversity; (**C**) Genus-level composition; (**D**) Differential species heatmap; (**E**) *Oscillospira* abundance; (**F**) *Ruminococcus* abundance; (**G**) *Coprococcus* abundance; (**H**) *Enterobacter* abundance; (**I**) *Parabacteroides* abundance; (**J**) *Lachnospiraceae_Clostridium* abundance. The results are expressed as mean ± SD, *n* = 4. The significance of the differences was estimated by one-way ANOVA, * *p* < 0.05, ** *p* < 0.01, *** *p* < 0.001.

Gut microbiota analysis demonstrated significantly higher alpha diversity (Chao1, Shannon indices, *p* < 0.05) in the rhLYZ group compared to both control and HEWL groups, indicating comprehensive enhancement of species richness and overall diversity (Figure 15A). Beta diversity analysis via PCoA based on Bray–Curtis distances revealed substantial deviation of microbial community structures in both HEWL and rhLYZ groups from controls (PCO1 = 56.38%) (Figure 15B). At the genus level, rhLYZ exhibited a distinct dominant flora structure characterized by unique enrichment of beneficial bacteria including *Oscillospira* (producing short-chain fatty acids/exerting anti-inflammatory effects), *Ruminococcus* (stabilizing intestinal barrier/degrading complex polysaccharides), and *Coprococcus* (butyrate-producing/maintaining homeostasis), (Figure 15C) while suppressing pathogens like *Enterobacter* (opportunistic pathogens). Conversely, HEWL primarily differed from controls through upregulation of inflammation-associated *Parabacteroides* and downregulation of commensal bacteria such as *Lachnospiraceae_Clostridium*. Regarding species composition, rhLYZ promoted colonization of butyrate-producing bacteria (e.g., *Coprococcus*) and barrier-enhancing species (e.g., *Ruminococcus*), whereas HEWL reduced key commensals like *Lachnospiraceae_Clostridium*. Collectively, rhLYZ enhances intestinal health by optimizing microbial structure and function, while HEWL may induce microbiota dysbiosis (Figure 15D–J).

## 4. Discussion

Recombinant human lysozyme (rhLYZ) and hen egg white lysozyme (HEWL) exhibit fundamental differences in physicochemical properties, cytoprotective effects, and promotion of intestinal development. Regarding physicochemical characteristics, rhLYZ demonstrates significant advantages: its enzymatic activity is twice that of HEWL (*p* < 0.05), which contrasts with prior research by Yang reporting HEWL possessed only one-third of rhLYZ’s activity. Our study further revealed that purified rhLYZ shares the same optimal temperature with HEWL, yet exhibits superior thermal stability within the 80–100 °C range [29]. Notably, the optimal pH values differed between purified rhLYZ and HEWL. This observation aligns with existing studies on HEWL [34] and oyster lysozyme [35], where optimal pH was shown to vary with salt concentration. Critically, rhLYZ’s optimal pH (6.0) more closely approximates the physiological intestinal environment, while its thermal stability at high temperatures (80–100 °C) significantly exceeds that of HEWL. This dual advantage enables rhLYZ to withstand the spray-drying process (70–100 °C) essential for infant formula production, providing a crucial safeguard for industrial applications [36].

Regarding cytoprotective effects, the two enzymes display opposing concentration-dependent responses. rhLYZ effectively alleviates LPS-induced damage to intestinal epithelial cells (*HT-29*) at low concentrations (1–10 μg/mL), whereas HEWL requires high concentrations (100–1000 μg/mL) to show efficacy. This difference suggests a “double-edged” nature of lysozyme function: low-dose rhLYZ repairs the intestinal barrier [37], while high doses may exacerbate inflammatory responses. This aligns with clinical observations—fecal lysozyme levels correlate positively with inflammation severity in Crohn’s disease patients, and Paneth cell-derived lysozyme can worsen colitis [32,33]. In contrast, HEWL’s requirement for high concentrations may increase potential inflammatory risks.

rhLYZ Promotes Intestinal Development and Maturation, Improving Nutrient Absorption Function. This animal experiment reveals that the promoting effect of recombinant human lysozyme (rhLYZ) on intestinal development in neonatal mice is highly consistent with core conclusions in the current field. The significantly enhanced body weight gain (Figure 8A,B) and optimized maltase-to-lactase activity ratio (Figure 8G) induced by rhLYZ directly echo the phenomena observed by Oliver, El Ratel et al. in piglet and broiler models: namely, lysozyme-induced improvements in gut morphology (such as increased jejunal villus height and reduced crypt depth) and nutrient absorption function [38,39,40]. From the gut microbiota-metabolite axis perspective, the rhLYZ-driven enrichment of *Ruminococcaceae* and *Oscillospira* (Figure 15C) significantly enhanced butyrate synthesis capacity [41]. This aligns completely with findings reported by Zou, Bastamy et al., wherein lysozyme increased butyrate-producing bacteria such as *Butyricicoccus* and *Ruminococcus* [40,42,43]. Butyrate efficiently supplies energy via beta-oxidation, providing the driving force for active nutrient transport across the intestinal epithelium [44,45]. At the immunoregulatory level, the observed upregulation of the *Defa* family (e.g., *Defa20/Defa21*) and the *Reg3g* gene (which targets and lyses Gram-negative bacteria), along with the dynamic expression of the chemokines *Cxcl5* and *Ccl28* (recruiting neutrophils and *IgA*-producing plasma cells) in this study, thus complements the mechanism described by Xiong wherein lysozyme enhances tight junction protein expression and blocks pathogen invasion [46].

This study reveals that the core mechanism underlying the functional limitations of HEWL in neonatal mouse models stems from its induction of immune dysregulation and host adaptability defects. Although HEWL can exert fundamental pathogen clearance by upregulating antimicrobial peptides *Reg3a/b/g* and complement genes *C3/C1RA* [47], its significant suppression of the Defensin family and *Tlr5* creates a defensive vulnerability against Gram-negative bacteria. This results in persistently elevated *Proteobacteria* abundance at 9.64%. More critically, HEWL’s broad suppression of adaptive immune genes—including *Cd79a* (a key *B-cell* activation factor) and *Ccr7* (a lymphocyte homing regulator) [48,49]—severely compromises *IgA* secretion efficacy and long-term immune surveillance. Such systemic immunosuppression may provoke chronic inflammation, ultimately counteracting its localized antimicrobial benefits.

While these findings appear contradictory to Bastamy et al.’s report of lysozyme reducing inflammatory factors [42], they profoundly reflect the decisive influence of host specificity on lysozyme functionality. Bastamy’s study, conducted in an avian model dominated by Firmicutes, allowed lysozyme to effectively activate the butyrate metabolic axis for anti-inflammatory effects. In contrast, within the Bacteroidetes-dominated murine gut microenvironment of this study, HEWL failed to enrich butyrate-producing bacteria (e.g., *Lachnospiraceae*), leading to insufficient metabolic support. Concurrently, structural differences between human-derived and non-mammalian lysozymes further amplified immunoregulatory deviations. This host-specific response divergence mechanistically aligns with lysozyme’s dual roles in colitis models [35,37]—where enzyme source variation (human rhLYZ vs. avian HEWL) and host microbiota baseline features *(Firmicutes* vs. *Bacteroidetes predominance*) jointly modulate metabolic efficiency (butyrate production) and immune tolerance thresholds, ultimately determining the biological trajectory of lysozyme effects.

**Figure 16:**
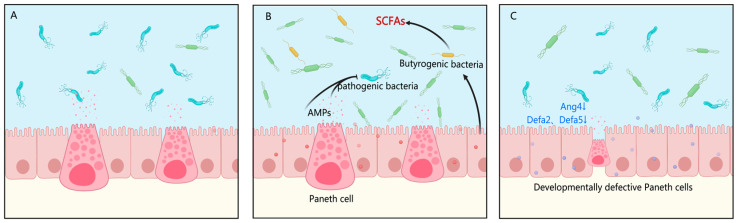
Differential mechanisms of rhLYZ and HEWL in regulating intestinal development in neonatal mice. (**A**) Control group: Depicts the baseline state of the intestinal milieu with normal levels of antimicrobial peptides (AMPs), Paneth cell function, and microbial communities; (**B**) rhLYZ group: Treatment enhances intestinal defense by upregulating AMPs (e.g., Defa2, Defa51, Ang4) and Paneth cell function. It concurrently promotes the expansion of butyrogenic bacteria, increasing SCFA production that supports barrier integrity and metabolic health, forming a virtuous cycle; (**C**) HEWL group: Treatment leads to developmentally defective Paneth cells and a suppressed AMP profile. This results in a failure to enrich butyrogenic bacteria and control pathogenic bacteria, ultimately disrupting intestinal homeostasis.

Based on the comprehensive findings, the superior efficacy of rhLYZ in promoting intestinal development can be attributed to its ability to remodel the gut microbiota structure and exert dual immunomodulatory and microbial regulation. Specifically, rhLYZ enriched butyrate-producing bacteria such as *Ruminococcaceae* and *Deferribacteraceae*, with the resulting butyrate supporting energy metabolism and barrier repair. In parallel, rhLYZ upregulated antimicrobial peptides (e.g., Defa family) and immune-related genes (e.g., *Reg3g*, *Cxcl5*, *Ccl28*), enhancing defense against pathogens such as *Proteobacteria* (Figure 16A,B). In contrast, HEWL induced immunosuppressive effects, notably suppressing the Defensin family and *Tlr5*, which weakened Gram-negative bacterial clearance and led to persistent *Proteobacteria* colonization (9.64%). Concurrently, HEWL failed to enrich key butyrate-producing taxa such as *Lachnospiraceae,* resulting in insufficient butyrate production and limited metabolic and barrier support, thereby constraining its promotive effects on intestinal development (Figure 16C).

The functional superiority of rhLYZ established in this study—driving a more balanced immune response and a healthier gut microbiota—naturally leads to the question of its practical applicability. Encouragingly, the path to industrial-scale production is well-supported. Previous work by our group has confirmed efficient rhLYZ expression using bovine mammary gland bioreactors [26,29], while other studies have highlighted the potential of microbial systems for scaled production [50]. Critically, our finding that rhLYZ possesses superior thermal stability (80–100 °C) directly addresses a key industrial requirement. When combined with its enhanced bioactivity at low concentrations and inherent human biocompatibility, these attributes position rhLYZ not only as a functionally superior candidate but also as a viable one for the next generation of infant nutrition. Ongoing optimization of expression systems will further solidify this promising outlook.

While this study demonstrates that early-life rhLYZ supplementation promotes intestinal development and beneficially reshapes the gut microbiota, several limitations should be acknowledged. First, the causal relationship between microbiota remodeling and improved intestinal morphogenesis remains unclear, and we cannot rule out whether the enrichment of butyrate-producing bacteria is a direct driver of maturation or a secondary consequence of an improved gut environment. Second, although we observed upregulation of antimicrobial peptides (e.g., Reg3g, Defa family) along with microbial shifts, the mechanistic link remains speculative in the absence of functional validation using knockout models. Finally, the long-term physiological relevance of the observed microbial changes—including the persistence of the altered community structure and the potential impact of low-abundance opportunistic pathogens such as Bilophila and Mucispirillum—remains unknown, as our study did not extend beyond the suckling period. These limitations highlight the need for future mechanistic and longitudinal investigations to fully assess the translational potential of rhLYZ in infant nutrition.

## 5. Conclusions

Our findings establish rhLYZ as a functionally superior alternative to HEWL in supporting intestinal development and homeostasis. The enhanced efficacy of rhLYZ stems from its optimized physicochemical profile—demonstrating greater enzymatic activity, thermal tolerance, and intestinal-compatible pH stability—fulfilling both physiological and industrial processing requirements. Functionally, rhLYZ orchestrates a dual regulatory response: it enriches butyrate-producing *Ruminococcaceae* to reinforce epithelial energy metabolism and barrier integrity, while simultaneously elevating defensin-mediated antimicrobial defense to restrict pathogens such as *Proteobacteria*. Crucially, rhLYZ exhibits pronounced host adaptability, circumventing the HEWL-induced immunosuppression and disruption of butyrate metabolic support that ultimately constrain its efficacy. This study demonstrates that recombinant human lysozyme (rhLYZ) holds substantial promise for industrial application in infant formula. Notably, rhLYZ exhibits superior thermal stability (80–100 °C), making it highly compatible with spray-drying processes used in formula production. Significant progress has been made in scalable production systems, including efficient expression in bovine mammary bioreactors and optimized microbial platforms. Together with its enhanced bioactivity, human bio-compatibility, and beneficial impacts on gut immunity and microbiota, rhLYZ represents a functionally advanced and industrially viable candidate for the next generation of infant nutrition.

## Data Availability

The original contributions presented in this study are included in the article. Further inquiries can be directed to the corresponding author.
